# Yellow Fever

**Published:** 1900-11-10

**Authors:** 


### YELLOW FEVER.

It has long been believed by some, although the idea has been ridiculed by others, that yellow fever can be conveyed by the bite of the mosquito, and that the immunity (more or less) enjoyed by those born and brought up in yellow fever localities is due to repeated inoculation from infancy upwards by means of these insects. The idea that the immunity was so maintained was strongly enforced by the well-known observation that even natives after prolonged absence from yellow fever districts are found on return to have lost some of their power of resistance. It is now stated that experiments made in Cuba have demonstrated the possibility of the virus being inoculated in this way. It is not without interest to note that, whatever the cause may be—whether it be the improved standard of sanitation which has followed on the occupation of the country by the United States or some other influence—the fatality of the cases of yellow fever which have occurred in Havana during the past two Septembers has been less than it has been for the last ten years during the same month.[1] The average death-rate lately among the cases has worked out at 25·68.

[1] Medical Record, October 20.

